# Group Psychoeducational Intervention for Grandparents of Young Children with ASD: An Open Feasibility Study

**DOI:** 10.1007/s10803-021-05189-0

**Published:** 2021-07-10

**Authors:** Rano Zakirova-Engstrand, Lise Roll-Pettersson, Kristina Andersson, Helena Larsson, Mara Allodi Westling, Tatja Hirvikoski

**Affiliations:** 1 Department of Special Education, Stockholm University, 106 91 Stockholm, Sweden; 2 Habilitation & Health, Stockholm County Council, Stockholm, Sweden; 3 Department of Women’s and Children’s Health, Center for Neurodevelopmental Disorders at Karolinska Institutet (KIND), Pediatric Neuropsychiatry Unit, Karolinska Institutet, Stockholm, Sweden; 4 Center for Psychiatry Research, Region Stockholm, Stockholm, Sweden

**Keywords:** Feasibility, Grandparents, Psychoeducational intervention, Family systems

## Abstract

This initial open feasibility trial reports on feasibility and preliminary effectiveness of the manualized, group-based psychoeducational intervention for grandparents of preschool-aged children with ASD provided by the outpatient habilitation services in Stockholm, Sweden. One hundred and twenty non-custodial grandparents participated in a 6-h intervention program. The study demonstrated good feasibility: 114 (95%) grandparents completed both pre- and post-intervention measures and evaluations and reported high intervention acceptability. The results also indicated that grandparents increased their knowledge about ASD from pre-intervention to post-intervention, gained skills about strategies of supporting their grandchildren and adult children, and appreciated the opportunity to meet and share experiences with other grandparents. Follow-up with a randomized controlled trial design is needed to firmly establish efficacy of this intervention.

**Supplementary Information:**

The online version contains supplementary material available at 10.1007/s10803-021-05189-0.

## Introduction

Autism spectrum disorder (ASD) is a neurodevelopmental condition characterized by restricted, repetitive behaviours and interests as well as impairments in social communication and social interaction across multiple contexts (American Psychiatric Association, 2013). ASD is also characterized by heterogeneity in both its symptomatic presentation and impairment (Klin & Jones, 2018). Co-existing conditions may also include learning disability, eating problems, sleep disorders, behavioural problems, ADHD, depression and anxiety (Gillberg, 2010), which can adversely affect well-being of the child’s family members. Parents to children with autism report elevated levels of stress (Hastings, 2008). For instance, in Sweden Renhorn et al. (2019) showed that parents of children with neurodevelopmental disorders are expected to know how to navigate the complexity of formal support systems; however, they are often left without adequate support in coordination of care for themselves and their children, causing a feeling of frustration, increased care burden and high parental stress. In this context, support from extended family members—grandparents—can become vital for energy and well-being in the family (Hastings, 2008).

Existing research has demonstrated that traditional grandparents’ role in the family can be invaluable for children with ASD and their parents. A recent review of peer-reviewed publications on the traditional, non-custodial grandparents of children with developmental disabilities, including ASD, by Novak-Pavlic et al. (2020) demonstrated that these grandparents play an active role in families by providing emotional, financial and instrumental support to their adult children and grandchild with a disability. For instance, Prendeville and Kinsella (2019) in their study with nine families of school-aged children with ASD in Ireland found that grandparents played an important role in strengthening the family system by providing respite care for their grandchildren. This study also emphasized a particular role that grandfathers had in their family system—becoming “a father figure” in those situations when the grandchild’s father did not participate in the child’s life, or having a “calming role” when dealing with the grandchild’s challenging behaviour (p. 744). In an exploratory study from the United States, Hillman et al. (2016) investigated first-person experiences of grandparents of children with ASD and found that the grandparents made significant accommodations to support their families, e.g., they combined or moved households to provide grandchild care; they provided transportation and babysitting or helped financially by paying for grandchild’s educational services, occupational and speech therapy, therapy based on applied behaviour analysis (ABA), or for legal support and advocacy. Given this important role of grandparents in families living with autistic children, supporting grandparents may strengthen the whole family system.

However, grandparents may also face multiple and heterogenous challenges related to their role as grandparents to a child with a developmental disability (Kahana et al., 2015). They can experience a variety of mixed feelings and emotions when their young grandchild is diagnosed with a disability or a chronic condition: on the one hand, they may feel much happiness about a child who has entered their family; on the other hand, they may experience double grief or even triple grief—for their own adult child, for the grandchild, and their own sorrow with feelings of guilt and injustice (Dolbin-MacNab et al., 2019; Dougherty, 2009; Kahana et al., 2015; Klasen McGrath, 2008). Grandparents can also experience feelings of gratitude that their grandchild could get a diagnosis and appropriate support; besides, many grandparents show their readiness and willingness to stand by and relieve their adult children of daily care for the young child with disability. Previous studies with grandparents of children with ASD described their experiences as “striving for answers”: they often struggled with the questions on how much they could help their adult children; or looking for answers on a probable cause of the disorder, “with their focus often being projected towards in-laws, medical failings, or the triple MMR vaccine” (Margetts et al., 2006, p. 571).

Furthermore, several studies reported that grandparents may have their own needs in relation to their grandchild with autism. A qualitative study with custodial grandmothers (i.e., primary caregivers) of grandchildren with disabilities, including autism, demonstrated that grandparents had the greatest need in learning how to deal with the grandchild’s aggressive behavior and/or language difficulties; they also had informational needs on their grandchild’s diagnosis and community services such as clubs or summer camps (Gallagher et al., 2010). The accumulated research evidence on traditional, non-custodial grandparents of children with ASD have indicated somewhat similar findings: among the most frequent needs expressed by grandparents were information about the child’s ASD and learning how to manage grandchild’s challenging behavior (Engstrand et al., 2020; Hillman, 2007; Hillman et al., 2017; Prendeville & Kinsella, 2019). A recent quantitative study showed that besides the identified needs highlighted above, the traditional grandparents expressed the need to learn how to adapt play or recreation activities for their grandchild and how to communicate with their grandchild’s teachers or other professionals (Zakirova-Engstrand et al., 2020). Other grandparents’ needs reported in the literature included a need for social support to manage stressors in the family (Hillman, 2007). In a later study, Hillman et al. (2014) found that the majority of grandparents coped fairly well with their grandchild’s ASD, although they expressed much worry for their adult children. However, the same study reported that 12% of grandparents had difficulties in coping with their grandchild’s ASD, and 29% responded sought professional help including turning to support groups. To enable them to manage their feelings and to navigate family events around their young grandchild, grandparents would benefit from clinical interventions or other support services to learn about ASD, to acquire skills and strategies on how to address grandchildren’s challenges and to improve supportive relationship with their adult children. However, there is a dearth of interventions focused on grandparents in comparison with the child’s nuclear family members—parents (Dolbin-MacNab et al., 2019; Kirby, 2015); moreover, it appears that there are virtually no studies reported on the effectiveness of interventions designed specifically for traditional (non-custodial) grandparents of children with developmental disabilities, including children with ASD (Novak-Pavlic et al., 2020; Zakirova-Engstrand et al., 2020). Furthermore, research evidence on services needs for caregivers of children with ASD to improve their quality of life is still scarce, and evaluation of effectiveness of already existing treatments and interventions for this group has been acknowledged as a research priority in the field of autism (Cervantes et al., 2020; Interagency Autism Coordinating Committee [IACC], 2017). Thus, evaluating existing intervention for grandparents could be a feasible way of contributing to the limited evidence base within autism service research, especially focusing on grandparents. The gained knowledge could be used to guide the design of future interventions for grandparents of young children with ASD.

### Theoretical Framework

To address the complex needs of children with ASD and to support their families, there is an increasing focus on providing family-centered care for the entire family (Dunst et al., 1994; Hodgetts et al., 2013). Family-centered approach encompasses provision of services and supports for children with ASD and their families across various sectors: medical, educational, social and community, aiming at “empowering families with the knowledge and skills to make the best choices for their children and for the family unit, as a whole” (Prizant, 2009, p. 30). The overarching theoretical framework for the intervention for grandparents of children with ASD reported in the present study is based on system theory thinking; more specifically, the study derives from the principles, assumptions and propositions advanced by family-system theory (Cox & Paley, 1997) and the bioecological model of human development (Bronfenbrenner, 1979; Bronfenbrenner & Ceci, 1994). Family-systems theory views the family as a social system—a complex network of shared relationships with its unique features, goals and needs—where each family member as an open system interacts with the child and other family members as systems (Cox & Paley, 1997; Dalmau et al., 2017). It examines how various family factors and processes can co-vary or influence each other to produce changes for individual family member or family as a whole over time (Wachs, 2000). The bioecological model of child development helps to understand how various environmental factors can influence family processes and outcomes when families engage in complex, reciprocal interactions with people in other settings of the meso- or exosystem level that can directly or indirectly affect the developing child (Bronfenbrenner, 1986). It is important to emphasize that some components of the intervention were also informed by the biopsychosocial model of disability as a fundamental framework for the International Classification of Functioning, Disability and Health (ICF; World Health Organization [WHO], 2001) with the emphasis on child’s strengths and abilities in everyday functioning rather than deficits associated with ASD (Bölte et al., 2019).

### Psychoeducation-Based Intervention Programs for Families with ASD

Many family-centred practices have shifted their focus from providing support to families on individual basis to organizing psychoeducational group-based training courses for parents to teach skills and knowledge about ASD (Pillay et al., 2011). The group format provides not only information-based training or intervention programs, but also offers support and access to others in similar situation to share their experiences. One of the psychoeducational group-based programs, primarily developed to support parents of young children with ASD, described in the literature is the EarlyBird designed by the National Autistic Society (NAC) in the UK (Shields, 2001). This three-month program provides post-diagnostic support for families of children younger than 5 years (Palmer et al., 2020) and aims at improving child social communication skills and reducing problem behaviour by educating parents about autism and building their competence and confidence to meet the needs of their children (Shields, 2001). The program was designed to include six families who participate at eight weekly group sessions each lasting three hours and three home visits by practitioners who observe parent–child interactions. During group sessions, the video clips from home visits are used to provide feedback; also, parents are encouraged to share experiences and support each other (Palmer et al., 2020; Shields, 2001). The feasibility studies conducted in various cultural contexts of this program have demonstrated parents’ high levels of satisfaction with the courses, their usefulness as well as positive outcomes in parents’ learning how to manage children’s behaviour or their own stress (Dawson-Squibb & de Vries, 2020; Dawson-Squibb et al., 2019; Palmer et al., 2020). Another program developed and manualised in the UK by Wright and Williams (2007) is known as Autism Spectrum Conditions—Enhancing Nurture and Development (ASCEND; Pillay et al., 2011). This psychoeducational skills training course was designed for parents of school-aged children (4–18 years) with autism with the purpose to educate them about ASD and to teach skills on how to manage their child’s challenging behaviour. Parents had also opportunities to exchange their experiences with each other. The training course consisted of 11 weekly sessions lasting 2 h each. The evaluation of this intervention program showed enhanced parental confidence and understanding of difficulties associated with ASD, satisfaction with the course and improved child behaviour as reported by parents (Pillay et al., 2011). A third example of extensive psychoeducational skills training program that has been used with parents of children with autism is the Stepping Stones Positive Parenting Program (Stepping Stones Triple P [SSTP]; Sanders et al., 2004) developed in Australia. The SSTP is child- and family-centred program that employs a public health approach to parenting with five levels of intervention—ranging from low-intensity to high-intensity—designed specifically for families of children with developmental disabilities aged 2–8 years, and it teaches parents effective management strategies to deal with child challenging behaviour and development issues (Sanders et al., 2004; Tellegen & Sanders, 2013; Whittingham et al., 2009). The program is a modification of the Triple P—Positive Parenting Program that was originally designed for parents of typically developing children “who have or are at risk of developing behavioural or emotional problems" (Sanders et al., 2004, p. 265). Several studies evaluated the efficacy of the program with parents of children with ASD and reported a considerable decrease in children’s behavioural problems, a decrease in dysfunctional parenting style, improvements in parental adjustment, parenting confidence, and parental relationships with their partners (Tellegen & Sanders, 2014; Van Voorhis et al., 2015; Whittingham et al., 2009). The Triple P intervention program is the only one of the above-mentioned programs designed to address the needs of grandparents who provide care to their grandchildren (Kirby & Sanders, 2014a, 2014b). It has been suggested that a less intensive format of the program could be used as a possible way of supporting non-custodial grandparents parallel to parent support (Kirby, 2015; Kirby & Sanders, 2014a). However, grandparents of children with autism were not included in these studies.

Various outcomes measures assessing parental knowledge of ASD at baseline and at post-intervention were used across the extensive psychoeducational programs outlined above. For instance, studies evaluating the NAS Early Bird parents support program utilized the following instruments: (1) the four-item subscale “Knowledge About Autism” as part of the Autism Parent Questionnaire (APQ; Anderson et al., 2006; Palmer et al., 2020); (2) the 10-item knowledge of autism section included in the Parent Involvement Questionnaire (PIQ; Solish & Perry, 2008), and finally (3) data from qualitative interviews that provided evidence on parents’ increased knowledge of autism (see Dawson-Squibb et al., 2019 for a review). Furthermore, for their study that evaluated the ASCEND program, Pillay et al. (2011) developed and used a 22-item Parent learning questionnaire that covers the main areas of difficulties associated with ASD. No measures assessing knowledge of autism were reported by studies that evaluated the efficacy of the Stepping Stones Triple P program, although this program was the only one that included an additional parent outcome measure—the quality of parents’ relationships with their partners. Nevertheless, none of these empirically supported programs described above investigated the quality of relationships with their extended family members such as child’s grandparents. Previous research has showed that disagreements in views about a child with autism between grandparents and the child’s parents could potentially lead to intergenerational conflicts and poor relationships that might result in grandparents’ lesser involvement in the lives of their grandchild with ASD (D’Astous et al., 2013; Glasberg & Harris, 1997). Kirby (2015) argues that the main goal of interventions designed for non-custodial grandparents should be teaching them strategies aimed at developing communication skills in order to facilitate good relationships with their adult children. However, to date there is no available literature reported on similar programs designed specifically for grandparents of young children with ASD (Zakirova-Engstrand et al., 2020), although frequently recognized as an important area of development by interest organizations (Hirvikoski et al., 2015). The purpose of this paper is to contribute to the literature by describing and reporting findings from the evaluation of a one-day intervention provided to grandparents of young children with ASD by healthcare habilitation services in Sweden.

## Research Questions and Hypotheses

The present study aimed to assess feasibility and preliminary effectiveness of the group-based psychoeducational intervention program offered to grandparents of young children with ASD in a publicly funded outpatient health and disability (habilitation) services context. The study sought answers to the following research questions: (1) To what degree are grandparents of young children with ASD satisfied with the course/intervention? (2) Is participation in a psychoeducational intervention about ASD associated with increased knowledge among grandparents of young children with ASD? It was hypothesised that (i) participants satisfaction with the course/intervention (i.e., treatment satisfaction as part of the feasibility of the intervention) would be moderately high across participants; (ii) participants would significantly improve from pre-intervention (T1) to post-intervention (T2) on ASD knowledge (i.e., preliminary treatment effectiveness).

## Method

### Design and Setting

This open feasibility study was part of a larger research project aiming at investigating needs and experiences of being a grandparent of preschool-aged children with autism within the context of the Swedish support system. The study was carried out in collaboration between the Autism Center for Young Children (ACYC) at Habilitation & Health, Region Stockholm, the Department of Special Education, Stockholm University, and the Center for Neurodevelopmental Disorders at Karolinska Institutet (KIND). Data was collected at the ACYC Habilitation & Health unit located in Stockholm. The main target group of this unit is children up to 7 years of age (at referral to the center maximum of 4 years) including children with or without co-existing intellectual disability (of different severity levels), and their parents as primary caregivers. The catchment area is all of Stockholm County comprising 25 municipalities with a total population of 2.2 million inhabitants. The ACYC provides psychoeducational training programs based on the principles of ABA aimed at parents of young children with ASD (Region Stockholm, 2019) ranging from introductory and follow-up courses (for those families whose children recently got the diagnosis) to thematically structured workshops, for instance, “Communication”, “Eating challenges”, “Problem behavior”, “Routines, rituals and stereotypical behaviors”, “Sleep and sleep hygiene”, “Toilet training”. In addition, parents also receive individual supervision from habilitation professionals. Alongside psychoeducational interventions for parents, the ACYC provides a support program for siblings of young children with ASD. One of the missions of the ACYC is to provide individual supervision to preschool personnel who deliver Early Intensive Behavioral Intervention (EIBI) to children at community-based preschool settings (Roll-Pettersson et al., 2016). Additionally, the ACYC offers a day-long introductory course for preschool personnel; besides, preschool teachers can also participate in the thematic workshops together with parents of children with ASD. Although not the center’s main mission, courses offered by the ACYC to children’s extended family members such as grandparents include one day long introductory course that aims at supporting the family system around the child with autism.^1^ Thus, the study used an open pragmatic design and applied a mixed-methods approach to assess feasibility of this intervention in an outpatient clinical habilitation context.

#### Ethical Considerations

**Fig. 1:**
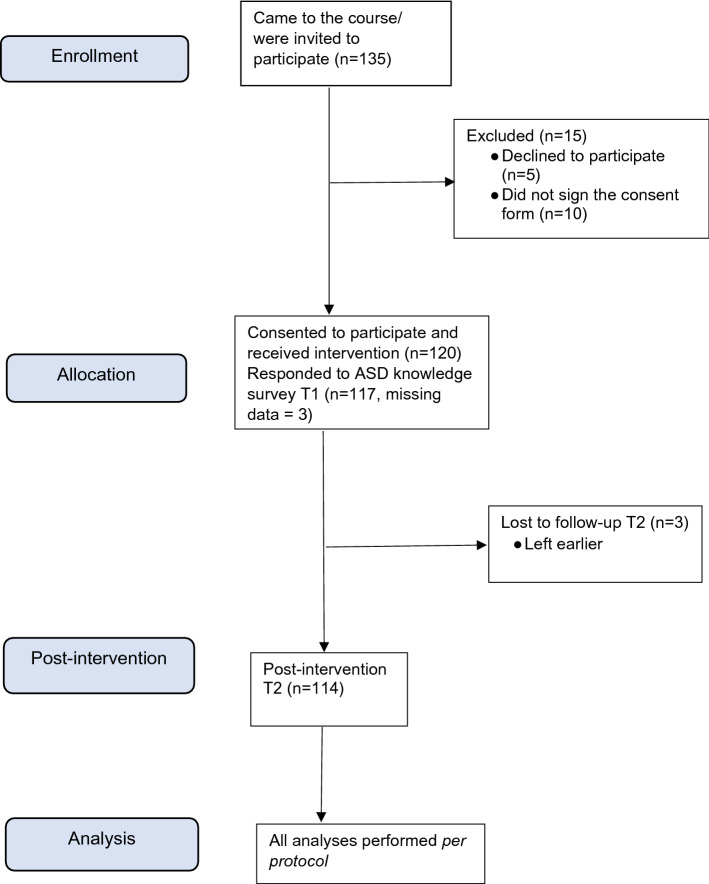
CONSORT diagram over the study participants

The study was approved by the Regional Ethical Review Board in Stockholm (reference number: 2017//286-31/5). To ensure the research ethics of the current study, we followed the ethical principles for medical research involving human subjects outlined in the Declaration of Helsinki as well as the principles of the European Convention on Human Rights and Biomedicine (Council of Europe (n.d.). The study also adhered to the regulations of the Swedish Authority for Privacy Protection (n.d.). The grandparents were first verbally informed about the aims of the study and their rights as research participants, i.e., that their participation was completely voluntary, that they could withdraw their participation at any time, and that obtained data would be fully confidential, and be used only for the research purposes. After that each grandparent received an envelope containing an information letter, a consent form and the questionnaires in paper form. The information letter described the aims of the project and the study’s procedure; it also informed about grandparents’ right to voluntary participation and their right to withdraw from the study at any time without any further explanation; the letter also informed the course participants that obtained data would be fully confidential and be used only for the research purposes. One hundred and twenty grandparents (n = 89%) agreed to participate (Fig. 1 Written consents were obtained from these grandparents; all data were included into analyses. To protect identity of participants and to maintain the confidentiality of the data, several measures were undertaken: numeric codes were assigned to each grandparent; data collected from the participants were analyzed on a group level and stored in password protected files on the university-maintained servers with secured backup; only two researchers – the first and the last authors – had access to the data.

### Intervention Description

The intervention program for grandparents with a preschool-aged grandchild with ASD is intended for grandparents whose grandchildren have been recently diagnosed with ASD and have started receiving treatment and services at the ACYC unit. The intervention was developed by the ACYC practitioners and has been delivered for more than a decade. The course was modified based on feedback gained from the group leaders and the course attendees in the past, as well as evolvement in knowledge about ASD and clinical practice, e.g., changes in ASD diagnostic criteria (DSM-5; APA, 2013). It is important to note that all modifications to the course were already done before the data collection began in this study.

The intervention was a manualized and highly structured course organized as one-day long session (from 9 am to 4 pm). The course was typically offered to 2–3 groups of grandparents per semester. Group sizes comprised 30–35 maternal, paternal and extended grandparents. The objectives of the course were: (1) to increase grandparents’ knowledge of ASD as well as difficulties and strengths associated with the disorder; (2) to provide information on how grandparents can help to support their grandchildren’s positive development; (3) to discuss grandparents’ role in supporting their adult children, and (4) to ensure peer support, i.e., to provide opportunities to exchange everyday experiences with other course participants. The latter involved group discussions, which is one of the most important parts of the course. The course content was organised around five main topic areas, such as (1) “The diagnosis of Autism Spectrum Disorder”; (2) “A different way of thinking and feeling”; (3) “Sensitivity to demands”; (4) “How we learn and how we can teach our children with ASD the best way”, and (5) “How families or parenthood is affected” (Table 1).

**Table 1:** Description of intervention content

Topic areas		Content
1	“The diagnosis of Autism Spectrum Disorder”	Lecture informs about the core characteristic features of ASD and about the current diagnostic classifications and the diagnostic criteria for ASD (based on the DSM-5 and the ICD-10) used in Sweden. Focuses on strengths and deficits that children with ASD may have. Stresses the fact that children with ASD are not homogenous in their behavior presentation. Film demonstration on difficulties and opportunities that children with ASD may present, followed by discussions in pairs (“Your reflections on the film”)
2	“A different way of thinking and feeling”	Lecture provides definitions about perception and cognition and informs about difficulties in perception among children with ASD when processing information. Focuses on sensory sensitivities that accompanies ASD; provides several examples on how children with ASD may be hypersensitive or hyposensitive to various stimuli, e.g., taste, sight, smell, sound, touch. Discussion in small-groups (“Share your experiences with each other on how your felt when you first learned that your grandchild got an ASD diagnosis”)
3	“Sensitivity to demands”	Describes how difficulties associated with ASD may affect the grandchild’s and his/her family’s functioning in everyday life. Focuses on how children with ASD might be negatively affected by their failure to respond to environmental demands and expectations. Informs about various strategies to help the grandchild to succeed in their response to environmental challenges. Focus on support given to child’s parents
4	“How we learn and how we can teach our children with ASD the best way”	Focuses on the principles of learning and describes the best strategies to teach children with ASD. Informs about the importance of functional assessment to choose the most appropriate interventions. Provides information on strategies to communicate with the grandchild with ASD
5	“How families or parenthood is affected”	Discusses the impact of having a child with ASD on parents and the whole family system. Discussion in small groups (“How can I as a grandparent support my (adult) child?”). Provides additional information for further reading in form of online links to various informational websites and online discussion forums

The choice of the course’s learning objectives and topic areas were based both on clinicians’ opinions and on existing scientific evidence that had laid the ground for development of the most recent version of the diagnostic classification DSM-5 (APA, 2013) that conceptualises autism as a spectrum, characterised by deficits in core features in two domains: social communication and presence of restricted, repetitive patterns of behaviour and interests. For instance, course participants learned about child’s difficulties with initiating or responding to social interactions; difficulties with transitions or hyper- or hyposensitivity to sensory stimuli. At the same time, the intervention utilised a strength-based approach to education for family members of children with ASD by “viewing the positive aspects of a child’s behavior, highlighting areas of competence and identifying areas that facilitate development.” (Steiner, 2011, p. 179). For instance, grandparents learned that ASD can be associated with cognitive strengths and skills described in the literature, such as exceptional attention to detail or memory for details, expertise in specific topics, strong visual-spatial skills, and creativity, and such traits as being honest and telling the truth (Baron-Cohen, 2017; Mahdi et al., 2018a, 2018b; Warren et al., 2021). Grandparents learned about how to create positive relationships with their grandchildren by playing together with them and following grandchild’s interests. Other characteristic features of the intervention were that course participants got information about a number of empirically validated educational strategies based on the principles of ABA to teach grandchildren to acquire skills (Steinbrenner et al., 2020). They also learned more about the impact of child’s ASD on parents’ well-being (Hastings et al., 2014; Hayes & Watson, 2013) as well as obtained information on effective grandparenting strategies.

In this study, the course for grandparents started with a short introduction on the main principles of work and mission of the ACYC when providing healthcare services to young children with ASD and their family members. The course included a lecture with a 32-slide PowerPoint presentation, pair-group and small group discussions, and a video film demonstration. Each slide was accompanied by written notes for the course trainer with the first slide stating objectives and expected outcomes. Standardized handouts for participants consisted of hard copies of every presentation slide including the program. Course participants were encouraged to actively participate in the lecture and group discussions. There was one occasion for a pair group discussion and two occasions for small group discussions during the entire session. During the morning session, the grandparents were instructed to discuss in pairs the content of the short video film they had watched as a whole group and then to relate it to their own experiences. The film by G. Gerland is based on interviews with the parents of individuals diagnosed with ASD who shared their stories about difficulties they encountered and strategies they used to tackle these difficulties. For the small group discussion, all grandparents were randomly divided into six small groups placed in different rooms. The grandparents had an opportunity to share their experiences with each other on how they felt when they first learned about their grandchild’s ASD diagnosis. In the afternoon session, the discussions in small groups were structured around one topic: “How can I as a grandparent support my (adult) child?”. The course trainers were not present during these group activities to maintain the grandparents’ confidentiality and privacy. After discussions in small groups, grandparents shared their reflections with the whole group with the help of the course trainers who facilitated the discussion. At the end of the course, grandparents received information on useful websites they could independently access to learn more about autism, about legislation that regulate services for people with ASD, and about online discussion forums for families of children with ASD in Sweden. After that the participants were invited to complete an evaluation form following an informed consent process.

#### Course Trainers

Professionals who provided training at the ACYC Habilitation & Health unit were social workers, psychologists, special educators, speech and language pathologists with extensive experience of working with family-centered intervention programs. Each psychoeducational session was led by two trainers. In this study, the course trainers’ competence was regarded to be high as both trainers had actively taught the course for the last three years. Each trainer had an extensive experience of teaching this particular course as well as other courses as part of the family-centered intervention program delivered at the ACYC.

### Procedure and Participants

Grandparents of young children diagnosed with ASD obtaining treatment at Habilitation & Health participated in the study that took place at four full-day psychoeducational workshops held in March, May, October and November 2017. Grandparents were recruited following information that was disseminated to parents who engaged in therapeutic supports at the ACYC. Information about the study was provided to parents during their first introductory course offered to parents of young and newly diagnosed children with ASD. In addition, information about the course as well as a brief information about the study were presented in the Habilitation & Health’s digital course catalogue that both parents and grandparents had access to. In the catalogue, the course for grandparents was listed together with other courses designed for parents, siblings or preschool personnel. Parents had the opportunity to sign up their own parents or parents-in-law for participation in the course on behalf of their child’s grandparents *after* obtaining their consent. Grandparents could choose the most convenient date for their participation in the course and the study. Parents signed up grandparents to the course either by phone or by sending an email to the course administrator two weeks before the course started. Importantly, habilitation professionals usually ask parents to sign up the child’s grandparents for participation in the course as the ACYC needs parents’ permission for other significant people around the child to be able to access support services.

To participate in the study, grandparents who signed up for the course were invited to come to the training venue 40 min before the course started (i.e., by 8.20 am). As an incentive for participating in the study, the grandparents were invited for breakfast in the morning and fruits and refreshments at the end of the day. Upon arrival and after the breakfast, 135 traditional grandparents who came to participate in the course were invited to take part in the study. All participants were traditional (i.e., non-custodial) grandparents (Table 2); of them 75 (63.3%) were grandmothers and 43 (36.4%) were grandfathers. Regarding relationship to their grandchild, 36 (30.5%) were maternal grandmothers; 21(17.8%) maternal grandfathers; 35 (29.7%) paternal grandmothers, and 18 (15.3%) paternal grandfathers. Six grandparents (5%) described themselves as step-grandparents; two participants (1.7%) did not specify their relationship to the grandchild. The majority of grandparents reported being retired (n = 72, 60.5%), and more than a half had a graduate university degree (n = 65, 55.6%). The overwhelming majority of the participants were Swedish-speaking (90.1%); Seventy grandparents (n = 70, 62.5%) reported living in a large city. Almost 85% of the respondents reported their health condition as “good”. Of 119 grandparents (missing data n = 1), nine (7.6%) reported having two grandchildren diagnosed with autism. As reported by their grandparents, the grandchildren’s age ranged between 2 and 6 years; the mean age was 4.07 years (*SD* = 0.98; missing data n = 1); of 124 grandchildren (missing data n = 5), 94 (75.8%) were boys and 30 (24.2%) were girls. A more detailed description of grandparents’ sociodemographic characteristics is presented elsewhere (reference is deleted to maintain the integrity of the review process).

**Table 2:** Sociodemographic characteristics of study participants (n = 120) at baseline (pre-intervention)

Grandparent characteristics	N	(%)
Age (data missing = 2)		
40–55	5	4.2
56–65	45	38.1
66–75	63	53.4
Older than 75	5	4.2
Gender (data missing = 2)		
Women	75	63.6
Men	43	36.4
Relation to grandchild (missing data = 2)		
Maternal grandmother	36	30.5
Paternal grandmother	35	29.7
Step maternal grandmother	1	0.8
Step paternal grandmother	2	1.7
Paternal grandfather	18	15.3
Maternal grandfather	21	17.8
Step maternal grandfather	3	2.5
Other	2	
Mother language (data missing = 9)		
Swedish	100	90.1
Other European	6	5.4
Non-European	5	4.5
Education (data missing = 3)		
High school	11	9.4
Upper high school	41	35.0
University or university college	65	55.6
Employment (data missing = 1)		
Full-time work	18	15.1
Part-time work	12	10.1
Hourly work	1	0.8
Owns a company	2	1.7
Unemployed	2	1.7
Retired	72	60.5
Retired, but working part-time	12	10.1

### Measures and Instruments

The following questionnaires were used in the study: (1) the demographic survey, (2) the satisfaction with the course scale, and (3) the pre- and post-test ASD knowledge (pre-and post-tests were identical) scale.

#### Demographic Survey

The demographic survey was a modified and translated version of the demographic section of the Needs Survey for grandparents of children with disabilities developed by Dougherty (2009). It consists of 11 questions and asks participants to indicate their gender, age, mother tongue (as proxy for ethnicity), level of education, employment, perceived health, urban or rural setting of grandparent residence, and geographic proximity of grandparent residence to grandchild with ASD. The survey also asks questions on grandchild’s age and gender.

### Feasibility Measures

#### Treatment Fidelity

During the preparation phase of the current study, the first author attended one course session prior to the actual study began with the purpose to observe and collect information on trainers’ adherence to intervention protocol as well as on participants’ attendance and participation in the course activities. Observations showed that trainers followed rigidly the course manual, while the participants followed all course assignments. Besides, following the intervention’s protocol, at the end of the course all participants were given the same course evaluation form in the same room. The participants filled in the form under supervision of the course trainers. At this stage, the first author rated fidelity on a 5-item checklist (each item corresponding to 5 topic areas): “0 = not covered”; “1 = covered” for the entire session. The trainers covered all five (100%) key intervention components as per protocol. Based on these results, the research team decided not to assess treatment fidelity of intervention sessions (n = 4) that followed after the preparation stage.

#### Completion Rate

Completion was calculated as the number of grandparents who completed the intervention divided by the number of grandparents who consented to participation in the study. Benchmark for acceptable completion rate in a clinical setting for participants completing the whole course was set on 80%.

#### Treatment Satisfaction

To assess participants’ satisfaction with the intervention, the Course Evaluation form was used. The evaluation form was developed jointly by the research team and two senior course trainers who actively participated in its design and approved all items. The instrument is a survey consisting of 10 statements and one open-ended question that was specifically designed for this study. Four responses to the Likert scale items ranged from “0 = not at all”, “1 = unsure”, “2 = yes, partially” to “3 = definitely yes”. The scale measures grandparents’ satisfaction with the course on three dimensions: items 1–6 target grandparents’ appraisal of the content of the course as well as acquisition of knowledge and skills; items 7–8 evaluate helpfulness of sharing of experiences during group discussions, and items 9–10 evaluate whether gained grandparents would recommend this course to other grandparents and satisfaction with the course in general. The open-ended question asks the course participants whether they wanted to provide any additional comments. The Course Evaluation form was the same for all participants. The study participants were asked to fill in the form in the same room under supervision of the course trainers. In this study, the internal consistency of the scale (Cronbach’s alpha) was 0.76. An overall evaluation score was generated based on the mean rating of all ten statements.

#### Preliminary Efficacy Measure

To assess preliminary effectiveness, the ASD Knowledge Survey was used to collect pre- and post-intervention data. The survey consists of 20 items and was developed specifically for this study, items were partly based on the course content in consultations with the course trainers, and partly based on the items included in the Placement and Services Survey (PASS; Segall & Campbell, 2014). The PASS is a modified version of the Autism Inclusion Questionnaire (AIQ; Segall & Campbell, 2012) developed to assess factors affecting educational placement of students with ASD. It consists of several sections including the 10-item Knowledge scale. This scale is a condensed version of the Knowledge questions section consisting of 15 items included into the AIQ that demonstrated the psychometrically strong results with Cronbach’s alpha of 0.83 (Segall & Campbell, 2012). For the present study the permission to use the Knowledge items included in both questionnaires was obtained from Dr. M. Segall (personal communication, May 9, 2016) by the first author. Seven items from the PASS scale were included in our scale (see Table A, Online supplementary resource). Following Dr. Segall’s recommendations, we revised and modified some of the Knowledge items included in the PASS and the AIQ scales for a better fit for our study. While one item included in our survey retained its original wording in the Knowledge scale section in the PASS—*Children with ASD are very similar to one another*, several modifications were made to other six items, e.g., the original items *ASDs are developmental disorders* and *Medication can alleviate the core symptoms of ASDs* were modified into *Autism spectrum condition (ASC) is a neurodevelopmental condition* and *There are medications that help treat the core symptoms of autism*, respectively. In addition, we modified one item in the Knowledge questions in the AIQ based on the current diagnostic criteria reflected in the DSM-5 (APA, 2013)—the original item *The core deficits in ASDs are impaired social understanding, language abnormalities, and impaired sensory functioning* was replaced with the statement *Children with ASD have difficulties in two areas: (1) social communication and interaction and (2) restricted, repetitive behaviors, interests or activities*.

As mentioned earlier, other items included in the Knowledge scale in this study reflected the content of the intervention; they were generated based on accumulated empirical research findings on the situation of families of young children with ASD (e.g., *Stress is more common among parents of children with ASD than among parents of children without disability*; Hayes & Watson, 2013) or established evidence-based educational practices reported in the scientific literature (e.g., *Use of visual supports or objects can ease communication with children with ASD*; Steinbrenner et al., 2020). Similar to the AIQ and the PASS (Segall & Campbell, 2012, 2014), responses to the scale items in the present study were rated as “true”, “false”, and “I don’t know” with 1 point given for correct answer and higher total scores indicating better knowledge about ASD (see Table A, Online supplementary resource). In our data, the internal consistency of the scale (Cronbach’s alpha) at baseline was 0.75.

#### Data Analysis

Demographic data were analyzed descriptively using the SPSS software program (version 26.0). Feasibility of the intervention was evaluated both quantitatively and qualitatively. For the quantitative evaluation of data, frequencies and means for each item in the Course Evaluation form were calculated using descriptive statistics. Open-ended responses were analyzed using an inductive approach to thematic data analysis when themes were derived empirically from the data (Ryan & Bernard, 2003). These responses were extracted into a separate file with an additional variable—relation to grandchild—to match each response. Identification of themes involved reading the material several times and marking regularities in data with different colored pens. Reoccurring words and phrases in each response were coded. The codes were grouped into sub-themes and then organized into themes. The first and the last author discussed emerged themes and subthemes until they reached the consensus. Thereafter, quotations illustrating main themes were translated from Swedish into English.

To assess differences in scores on the ASD knowledge from Time 1 (pre-intervention) to Time 2 (post-intervention) we conducted a one-way repeated measures ANOVA (rmANOVA) as a within-subjects repeated measure factor. Missing data were treated by pairwise exclusion. The sphericity assumption was assessed by SPSS using Mauchly’s test; to compensate the assumption violation, we used the Greenhouse–Geisser adjustment. The alpha level was set at ≤ 0.05.

## Results

### Feasibility

#### Completion Rate

A total of 120 grandparents participated in this intervention. Six participants did not complete the intervention (see Fig. 1). The attrition rate was low; 95% of the participants (114 out of 120) completed the course.

#### Treatment Satisfaction

The treatment satisfaction ratings (min. 0 max. 3) for each item are shown in Table 3. In general, the grandparents reported that the course had met their expectations (*M* = 2.71, *SD* = 0.68) and that they would definitely recommend it to other grandparents with a grandchild with ASD (*M* = 2.88, *SD* = 0.51).

**Table 3:** Means and standard deviations for each item in the Course Evaluation Form (range 0–3; n = 114)

Items within the three assessed aspects of intervention satisfaction		Mean	SD
*Course contents and knowledge and skills acquisition*			
1	My knowledge about ASD has increased	2.80	0.46
2	The content of the lecture today was relevant according to my experiences	2.76	0.48
3	I have gained a better understanding of my grandchild’s unique strengths	2.52	0.62
4	I have gained a better understanding of my grandchild’s difficulties	2.72	0.52
5	I have learnt more about the role that habilitation plays in provision of support to my (adult) child and my grandchild	2.61	0.50
6	I have learnt more about how I can support my child (grandchild’s parent)	2.53	0.59
*Experience sharing*			
7	It was helpful to be able to share experiences with other participants	2.82	0.38
8	It was helpful to take part of others’ experiences and hear their advices	2.73	0.46
*Perception of the course as a whole*			
9	I would recommend this course to another grandparent who has a grandchild with ASD	2.88	0.51
10	Overall, the course has met my expectations	2.71	0.68

Responses rating: 0 = not at all, 1 = unsure, 2 = yes, partially, 3 = definitely yes

The results of the qualitative analyses identified three main themes based on the grandparents’ responses to open-ended questions (n = 30; 26% of the participants had responded). These themes were: Appreciation and gratitude, Suggestions for improvement, and Wish for participation in a follow-up course. Each theme is briefly discussed below.

#### Appreciation and Gratitude

The analyses of the responses showed that grandparents greatly appreciated the course for its content and for the opportunity to participate in group discussions. For instance, “Thank you for a very rewarding day!” (a paternal grandmother); “The opportunities for group discussions were of great value!” (a maternal grandfather). In addition, many grandparents also praised the course trainers for their professionalism, e.g. “I just want to say that the course teachers were very good!” (a step-maternal grandmother). One grandfather expressed his appreciation for the course and praised the course trainers in a following way: “Thanks a lot for the course! It is rare when you have a chance to see such a competent and pedagogical presentation during one and the same session!”.

#### Suggestions for Improvement

Several participants provided a number of suggestions on how the content of the course could be improved. For instance, one grandmother suggested the course could have provided more concrete information on how communication with grandchildren with ASD could be facilitated: “It could have been good with examples on how, for instance, visual supports/iPad can make communication with the child easier. Maybe with the help of a short film [it would be] especially important for those [grandparents] who have difficulties to communicate with the child.” A paternal grandmother wished to learn how to talk to her grandchildren about autism. Another paternal grandmother expressed a need for information on such topics as “siblings” and “food” (“whether gluten-free and dairy-free diets can help”). A maternal grandfather suggested the course leaders should include guidance and discussions on how “to face ‘lack of knowledge’ of others around you “. Another grandfather (a paternal grandparent) suggested introducing “a deeper discussion about interaction between generations”. Interestingly, two grandparents thought that sessions for discussion in small group should be set up differently. As one grandmother wrote: “Make groups based on the child’s difficulties”.

#### Wishing for Participation in a Follow-up Course

The participants clearly expressed a desire to participate in a follow-up course to be able to share their experiences and get practical advices. For instance, a paternal grandfather wrote: “[I] would like to meet again to exchange experiences”; a paternal grandmother noted: “I would be delighted to take part in ‘an additional course’ [to] share my experiences, practical advices, ‘how did it go after that’—a follow-up of the first course”. Three grandmothers expressed wishing participate in a follow-up course to obtain relevant information about their grandchildren’s eligibility to get services and support at school/special school as their grandchildren get older. For instance, a paternal grandmother wrote:”It feels like a continuation [of the course] is needed with more advices about school and contacts with the Social Insurance Agency and social insurance companies”. A maternal grandmother succinctly noted:” The school: special school [for students with intellectual disability] [or] regular school + help”. Two other maternal grandmothers wished for an additional course with different formats to include two generations, i.e. children’s parents and grandparents, and to include adult siblings to the grandchild’s parents (i.e. the child’s aunts and uncles): “Arrange a joint course where children’s parents and the older generation are present so that they can discuss [things] and so on”; “It’s desirable to have a course for ‘relatives’—e.g. siblings to the parents who are a big part of the children’s lives”.

#### Preliminary Effectiveness

The results of the descriptive statistics and rmANOVA are presented in Table 4. Mean ASD knowledge at baseline was 14.07 (*SD* = 3.31) and at post-intervention phase was 17.64 (*SD* = 2.29). There was a significant effect for time, Wilks’ Lambda = 0.42, *F* (1, 111) = 152.26, *p* < 0.0005, multivariate eta squared = 0.58, reflecting a large increase in ASD knowledge for grandparents from pre- to post intervention. Additional analyses using a Mann–Whitney U test revealed no significant difference in ASD knowledge at post intervention between grandmothers (*Md* = 18.00, n = 72) and grandfathers (*Md* = 18.00, n = 40), *U* = 1274, *z* = − 1.02, *p* = 0.31, *r* = 0.1. Furthermore, no statistically significant differences in ASD knowledge at post-intervention were found between maternal grandparents (*Md* = 18.00, n = 57) and paternal grandparents (*Md* = 18.00, n = 53), *U* = 1420, *z* =− 0.60, *p* = 0.58, *r* = 0.05.

**Table 4:** Results of repeated measures ANOVAs from pre- to post-intervention

Outcome measure	Baseline/T1 Mean (SD) *n*	Baseline/T2 Mean (SD) *n*	rmANOVA T1–T2, *p* value, ŋ2 effect size
ASD Knowledge	M = 14.07	M = 17.64	*F* (1, 111) = 152.26
	SD = 3.31	SD = 2.29	*p* < 0.0005
	*n* = 112	*n* = 112	ɳ2 = .58

## Discussion

To our knowledge, this is the first study that assessed the feasibility and preliminary effectiveness of the psychoeducational group intervention for grandparents of young children with ASD in Sweden. This study demonstrated good feasibility and acceptability of the intervention: the results showed that grandparents significantly increased their knowledge about ASD. Moreover, the grandparents reported that they gained skills about various strategies of supporting their grandchildren and adult children, and had opportunity to meet other grandparents who found themselves in the same situation, and they were able to share their experiences. They perceived the course as helpful and reported they would strongly recommend to other grandparents who have grandchild with ASD. Data also revealed the high attendance rates in our sample throughout the day, despite that some of them had to travel long distances to participate in the intervention. As such, the study tested the intervention manual to demonstrate whether the intervention was helpful in an outpatient clinical habilitation context. The active components of this psychoeducational intervention focused on provision of quality information about ASD and its diagnosis, about effective, evidence-based practices for child with ASD, as well as information about strategies to support both grandchildren with autism and their parents. The intervention also included group discussions that provided grandparents with several opportunities for sharing their experiences with each other during the course.

As part of the feasibility of the intervention, the study assessed participants’ satisfaction with the intervention. Our findings confirmed the study’s first hypothesis—grandparents’ satisfaction with the course was moderately high across participants—which was also reflected in their responses to the scale’s last item “Overall, the course has met my expectations. The mean scores for the survey items 1–6, targeting grandparents’ evaluation of the course content and their perception of acquired knowledge and skills, showed the highest rating for item 1 “My knowledge about ASD has increased” (*M* = 2.80, *SD* = 0.46). Importantly, this finding aligns with the results gained from the analyses of the responses to the 20-item ASD knowledge scale, which is the primary outcome measure in the present study. The results of the rmANOVA showed that grandparents’ knowledge about ASD significantly improved from baseline to post-intervention; besides, the results from the additional analyses using the Mann–Whitney U test revealed no statistically significant differences in ASD knowledge at post-intervention between grandmothers and grandfathers, or maternal and paternal grandparents, indicating that all grandparents irrespective of their gender or relation to the grandchild could increase their knowledge of autism. These findings demonstrate preliminary effectiveness of the intervention, thus confirming our second hypothesis. These findings are important as they correspond to the reported needs in previous studies indicating a need of information about ASD as one of the most prioritized needs expressed by grandparents of children with autism (Hillman et al., 2017; Prendeville & Kinsella, 2019; Zakirova Engstrand et al., 2020). Moreover, previous research on psychoeducational approaches to interventions in neurodevelopmental disorders such as ADHD and Tourette syndrome—conditions that can co-occur with ASD—has indicated that providing educational information increases knowledge, positive attitudes and behaviors toward children with neurodevelopmental disorders (Nussay et al., 2013). Indeed, earlier studies found that grandparents who participated in educational or support groups for extended family members of children with disabilities not only reported change from negative to positive attitudes among grandparents to their grandchildren with disability, but also revealed that these positive attitudes including feelings of acceptance, hopefulness and involvement could sustain over time compared to their counterparts who did not attend such support groups (George, 1988; Schilmoeller & Baranowski, 1998). More recent studies showed that grandparents’ good knowledge of ASD and strong, positive relationships with their adult children were important factors affecting grandparents’ greater involvement with their grandchild’s life (D’Astous et al., 2013).

Schilmoeller and Baranowski (1998) discussed the importance of support groups for grandparents of children with developmental disability. In their study, grandparents reported that they would want to talk to other grandparents who had grandchildren with the same type of disability as they did; they also expressed a wish to talk to professionals who worked with their grandchildren. Our findings are in line with these previous findings: both qualitative and quantitative data from the satisfaction survey showed that grandparents perceived the course as helpful in terms of sharing experiences with other grandparents. Thus, grandparents cherished the provided opportunity to meet other grandparents who were in the same situation and to share their experiences with them. The study’s qualitative data also revealed that grandparents perceived the trainers’ professionalism as of high standard. This is not surprising as the trainers in this study not only had very good knowledge about autism but also had a vast clinical experience with children with ASD and their family members, which is one of the strengths of how this intervention is delivered within the frames of the broader support program directed to families. Decroocq et al. (2020) noted that trainers’ professional background, competences, and experience in evaluations and support of child with autism are important prerequisites for successful implementation and delivery of psychoeducational programs for families of children with ASD. At the same time, it is important to note that the intervention was evaluated in an open non-randomized study design, which does not allow us to rule out the possibility that the grandparents' positive evaluations of the program were associated with the performance of specific trainers delivering the course rather than with the intervention program itself. On the other hand, the study sample consisted of four separate workshops and the evaluations were similar across the workshops and different trainers.

Several authors have argued for an adoption of a more holistic approach to provision of formal support services to families of children on the autism spectrum (Galpin et al., 2018). In Sweden, a family-centered, strength-based approach to provision of services for parents and other family members of children with autism by habilitation units is a well-established practice (Hedberg et al., 2010). The application of the strength-based model of interventions with the focus on child’s abilities and potentials rather than the use of the deficit-based model is a hallmark of the intervention program described in this paper. Earlier studies that used the strength-based approach to parent education for children with ASD showed improvements in parent affect and enhancement of parent–child interactions, which could potentially contribute to decreased stress levels and increased hope and optimism among parents (Steiner, 2011). Future studies could explore an impact of this strength-based intervention model on grandparents’ well-being by studying grandparents’ level of distress as well as grandparent–grandchild interaction patterns at post-intervention.

Among other strengths of the intervention is its multimodality. Dolbin-MacNab et al. (2019) singled out several approaches to intervention for grandparents, namely, provision of emotional and social support through support groups; teaching new skills (skills-based training interventions); information and psychoeducation organized as structured psychoeducational seminars or workshops, and intergenerational engagement programs. A multimodal intervention that combines components from various approaches—psychoeducation, skills training, and support groups—can be effective to address grandparents’ heterogenous needs and challenges they face when grandparenting a child with disability (Dolbin-MacNab et al., 2019). The intervention program described in the present study utilized these components to address the multiple needs of grandparents of preschool-aged children with autism.

### Limitations

The present study has several limitations. The study had an open, non-randomized design, which did not include a comparison group. However, this was a feasibility study carried out in the real-world outpatient clinical setting where the intervention was delivered by the habilitation practitioners aimed at investigating whether the intervention was feasible in their clinical practice (Smith et al., 2007)—an approach described in the literature as “bottom up” approach to intervention, unlike randomized controlled trials (RCTs) conducted in specialized research centers, or the “top-down” approach to intervention research (Black, 1996; Jonsson et al., 2016). Craig et al. (2008) argue that randomization may not be necessary when evaluating feasibility and acceptability of interventions that have been already widely used in clinical practice. Furthermore, variables in the present study focused on participant satisfaction with the course/intervention, which could be distinctive of effectiveness studies designs when outcome variables tend to be more global and fewer in number in comparison to RTCs (Smith et al., 2007). Moreover, our sample may not have been entirely representative in terms of socioeconomic and other demographic variables such grandparents’ health status or ethnicity. For instance, most of the participants were retired and had a university degree; furthermore, as our previous study (Zakirova Engstrand et al., 2020) demonstrated, financial needs were the least prioritized area of needs for this group of grandparents. Although this finding could be explained by the potential effects of the Swedish social welfare system and high level of formal public childcare provision, a small number of participants with immigrant background and those who were still in the workplace does not permit generalizability of our findings to all grandparents in Sweden and/or other Western, high-income countries. It is likely that grandparents’ low educational background, immigrant status, as well as financial challenges they face may be associated with a lower level of general knowledge of ASD. Future intervention research involving more representative samples of grandparents selected by using random methods is therefore required. Nevertheless, our sample is highly representative for the grandparents who participated in the intervention offered by Autism Center for Young Children. In addition, the attrition rate was low-proportion of participants completing the whole course was high, adding to the representativeness of the data, which is another strength of the study.

Finally, one methodological limitation of the study should also be mentioned. When designing the ASD Knowledge scale, opinions and perspectives of traditional grandparents were not sought and the scale was not piloted on grandparents to inform further development of the items. Instead, we chose a different approach: we used the items from the Autism Inclusion Questionnaire and its modified version—Placement and Services Survey (Segall & Campbell, 2012, 2014)—the most psychometrically sound measure designed to assess ASD-specific knowledge identified in the literature (Harrison et al., 2017). In parallel with that, we generated additional items guided by five main components of the program manual based on the contemporary conceptualization of ASD as well as on established research evidence on treatment. Interestingly, previous studies (Anderson et al., 2006) that evaluated the effectiveness of the EarlyBird program for parents of preschool-aged children with autism in New Zealand used a similar strategy: based on the program’s manual these researchers developed their own questionnaire to measure parent outcomes—changes in knowledge about ASD, skills, and attitudes as a result of the program. We believe that given the purposes of our study—to test preliminary effectiveness of the group intervention for traditional grandparents—the results obtained from the self-report ASD Knowledge Survey could be viewed as the initial testing of the scale that could inform and be used in larger-scale effectiveness studies with this population in the future.

### Implications for Practice and Research

The findings from the current study contribute to the field by providing first evidence to support the use of the psychoeducational group-based grandparent intervention program by habilitation services. The study’s qualitative findings revealed that some grandparents expressed a need for more concrete examples of how they could interact with their grandchild with ASD with one grandparent mentioning a need for “deeper discussions about relationship between generations”. Indeed, as Kirby (2015) argues,

a key issue that needs to be further addressed clinically is whether parents and grandparents should attend parenting programs together. The benefits of parents and grandparents attending sessions together is that it provides a medium to discuss expectations, resolve conflicts, problem solve, and discuss potential issues of control and decision making in a safe mediated environment. (p. 3209).

Autism Speaks (2018) also emphasizes that the main concern that grandparents of children with ASD face is the wellbeing of their adult children who provide care for their children.

Although the intervention manual in the present study does provide useful advices or ideas on how grandparents could support their adult children and grandchildren with autism, practitioners at habilitation services could add several specific and concrete examples to the manual, thus meeting the support needs of this group of grandparents further. Some grandparents suggested a new course designed especially for siblings—other adult children that grandparents had. This result supports the findings reported by Miller et al. (2012) who described the impact their grandchild with disability had on other extended family members—aunts and uncles—who wanted to help but were not sure if they could do it in the correct way; grandparents in this study also disclosed that they found themselves in the middle of family tension and conflicts as all their adult children expected similar amount of attention given to them and their grandchildren irrespective of needs, and therefore, “expressed anger, jealousy and possessiveness about how grandparents spent their time” (p. 107).

Given grandparents’ wish for a follow-up course, professionals at the outpatient habilitation services may want to consider planning and designing a follow-up course for grandparents as part of a more comprehensive intervention program for grandparents with children with ASD to meet grandparents’ needs over time, i.e., at grandchildren’s school entry with recognizable changes in needs among children with ASD, their parents and extended family members. Ultimately, this could be viewed as an issue of resource allocation that would guide design and planning of the future courses. Besides, it would be instructive to note that the key mission of the family-centered intervention program at ACYC is directed to parents of young children with ASD as primary caregivers, and where grandparents’ role is viewed as providing instrumental and emotional support to their adult children and grandchildren with ASD. On the other hand, one could also argue that this intervention program delivered by Habilitation & Health in Stockholm, can be described as a low-intensity intervention that according to Sanders et al. (2014) follows the public health principle of “minimal sufficiency” defined as “a concept that refers to the selection of interventions aimed at achieving a meaningful clinical outcome in the most cost-effective and time-efficient manner” (p. 339). In research, studies employing both longitudinal and qualitative designs could elucidate how grandparents’ needs regarding knowledge of ASD knowledge and peer support can change over time thus providing additional important evidence necessary for designing efficacious psychoeducational intervention programs for this group of family members. In addition, future studies could examine the impact of the intervention model described in this study on grandparents’ well-being by investigating grandparent–parent relationship, and grandchildren’s social and behavioral outcomes.

## Conclusion

Overall, our results suggest the clinical utility of the manualized, group-based psychoeducational intervention to address the needs of grandparents of preschool-aged children with autism in the outpatient clinical setting. The study adds to the evidence for family-centered interventions in autism specifically designed and delivered for grandparents of young children with ASD in Sweden and internationally.

## Supplementary Information

Below is the link to the electronic supplementary material.

Supplementary file1 (DOCX 16 kb)

## Footnotes

1 At the moment this course is cancelled due to COVID-19 pandemic.
