# Supplementary material for: Group Psychoeducational Intervention for Grandparents of Young Children with ASD: An Open Feasibility Study
Source: J Autism Dev Disord. 2021 Jul 10;53(2):808–24. doi: 10.1007/s10803-021-05189-0 (PMC8272608; doi:10.1007/s10803-021-05189-0)
Supplement: Supplementary file 1 — Supplementary file1 (DOCX 16 kb) [file 10803_2021_5189_MOESM1_ESM.docx]

*Table A. Online supplementary resource*.

**ASD Knowledge Survey**

All questions had *True*/*False*/*I don’t know* as multiple-choice answers.

|  |  |
| --- | --- |
| 1. | Autism spectrum condition (ASC) is a neurodevelopmental condition (*) |
| 2. | Children with ASD are very similar to one another (*) |
| 3. | Children with ASD have deficits in two areas: (1) social communication and interaction and (2) restricted, repetitive behaviors, interests or activities (**) |
| 4. | Sensory experiences (e.g., taste, sound, touching, smell) are often perceived differently by individuals with ASD |
| 5. | Children with ASD are not interested in playing with other children |
| 6. | Children with ASD often have one or more strong interests (*) |
| 7. | Children with ASD often have a good memory for details |
| 8. | Children with ASD may often fail with simple everyday tasks despite trying their best |
| 9. | Children with ASD have an innate unwillingness to cooperate (socialize) with others |
| 10. | It is better to be most attentive to things that children with ASD are good at (and not to those things they have difficulties with) |
| 11. | Autism can be caused by a bad upbringing (*) |
| 12. | Telling off makes it easier for children with ASD to understand how they should do |
| 13. | Autism can be cured by training (*) |
| 14. | In order to develop optimally children with ASD need more structure and a lot of repetition |
| 15. | Interventions grounded in learning psychology have demonstrated effectiveness for teaching children with ASD (*) |
| 16. | There are medications that help treat the core symptoms of autism (*) |
| 17. | Stress is more common among parents of children with ASD than among parents of children without disability |
| 18. | Asking open-ended questions is a good strategy if one wants to ease communication with children with ASD |
| 19. | Use of visual supports or objects can ease communication with children with ASD |
| 20. | Extensive adjustments (for example, in regard to communication), will entail that the child with ASD will not be able to learn or function within the context of his/her environment. |

*Note*: (*) denotes the items from the *Placement and Services Survey* (PASS; Segall & Campbell 2014) in their modified form; (**) denotes the item from the *Autism Inclusion Questionnaire* (AIQ; Segall & Campbell 2012) in its modified form.
